# Development and validation of a food frequency questionnaire for national nutrition surveillance among Malaysian adolescents and adults

**DOI:** 10.1017/jns.2026.10119

**Published:** 2026-07-01

**Authors:** Kimberly Yuin Y’ng Wong, Ahmad Ali Zainuddin, Ruhaya Salleh, Lalitha Palaniveloo, Azli Baharudin, Noor Ul-Aziha Muhammad, Khairul Hasnan Amali, Sulhariza Husni Zain, Wai Kent Lai

**Affiliations:** Centre for Nutrition Epidemiology Research, Institute for Public Health, Malaysia

**Keywords:** Dietary assessment, Food frequency questionnaire, Habitual food intake, Nutrition epidemiology, Validation

## Abstract

Food habits vary across ethnic groups and geographical regions. However, validated dietary assessment tools accounting for such diversity remain limited. A semi-quantitative food frequency questionnaire (FFQ) was developed and validated to assess the habitual food intake of adolescents and adults across Malaysia. The 147-item FFQ was constructed using commonly consumed foods from five main ethnicities (Malay, Chinese, Indian, and Sabah and Sarawak indigenous groups) identified from national surveys. A cross-sectional validation study was conducted among purposively sampled healthy individuals aged 10–59 years from 16 administrative regions. Trained community nutritionists administered the FFQ to assess monthly intake, alongside a three-day dietary record and recall (3DRR) covering two weekdays and one weekend. Spearman’s correlation, Bland–Altman plots, and quartile cross-classification evaluated the agreement between the FFQ and 3DRR for energy, macronutrients, and selected micronutrients (Vitamin C, thiamine, calcium, and iron). Respondents (n = 361; 50.3% adults, 49.7% adolescents) were 50.4% female and represented five main ethnicities (range: 15.8–25.2%), with 60.4% from Peninsular Malaysia. Energy intake estimated by the FFQ (median: 2285 kcal) was significantly higher than by the 3DRR (median: 1785 kcal; Wilcoxon p < 0.001). Spearman’s correlation coefficients observed for energy (crude r = 0.31), and selected nutrients (energy-adjusted r range: 0.19–0.38), along with <10% of extreme quartile misclassification indicated acceptable ranking ability and agreement for most nutrients. Bland–Altman plots indicated no proportional bias for energy and macronutrients. In conclusion, the FFQ is a valid tool for assessing dietary intake within the multi-ethnic Malaysian population nationwide.

## Introduction

The rapid nutrition transition in Malaysia, coupled with rising obesity and diet-related non-communicable diseases (NCDs), highlights the urgent need to monitor population dietary patterns. The National Health and Morbidity Survey (NHMS) 2023 reported that two-thirds of Malaysian adults were overweight or obese, while 95% consumed fewer than five daily servings of fruits and vegetables.^(1)^ As the Malaysian Dietary Guidelines (MDG) 2020 have provided recommended intake targets, assessing population adherence remains crucial.^(2)^ Unhealthy dietary habits were estimated to account for majority of productivity losses due to premature deaths from cardiovascular diseases.^(3)^ Specifically, intake of excessive sodium, alongside inadequate whole grain, and insufficient fruit are leading dietary contributors to cardiovascular risk.^(4)^ Comprehensive data on habitual dietary intake is therefore essential to inform national nutrition policies, guide public health interventions, and support the development of evidence-based guidelines.

Despite the public health urgency, Malaysia lacks a validated dietary assessment tool capable of reliably capturing habitual intake across its multi-ethnic population for national surveillance. The 24-hour dietary recalls and weighed food records, despite providing detailed data but are resource-intensive, burdensome for participants, and impractical for large-scale surveys. Furthermore, single 24-hour recall cannot accurately quantify certain nutrient intakes, particularly those with higher day to day variability. While three-day records are commonly used, recording quality generally declines if the duration is extended further.^(5–7)^ In contrast, food frequency questionnaires (FFQ) are widely utilized in nutritional epidemiology to estimate habitual intake over extended periods and are highly feasible for nationwide monitoring. This is particularly relevant as long-term dietary habits share a more significant relationship with chronic diseases than single-day intake.^(8)^ However, existing validated FFQs in Malaysia are either population-specific or restricted to certain age groups.^(9–12)^ A culturally adapted FFQ reflecting Malaysia’s dietary diversity is therefore critical to assess adherence to recommendations, monitor trends, and support evidence-based nutrition policies.

Therefore, this study aimed to develop and validate a semi-quantitative FFQ for assessing habitual dietary intake among Malaysian adolescents and adults. As dietary behaviours in adolescents often mirror those of adults due to shared household and community food environments,^(13)^ a single FFQ provides a practical and efficient approach for large-scale surveillance. The tool was designed to capture the diverse dietary habits of the country’s five major ethnic groups (Malay, Chinese, Indian, and the indigenous populations of Sabah and Sarawak) using commonly consumed foods identified from the Malaysian Adult Nutrition Survey (MANS) 2014 and the Adolescent Nutrition Survey (ANS) 2017.^(14,15)^ FFQ validity improves with additional reference days of 24-hour recalls.^(16)^ In this study, the FFQ was validated against a three-day dietary record and recall (3DRR) to determine its relative validity for estimating energy and nutrient intakes, ultimately providing a feasible instrument for nationwide dietary monitoring.

## Methodology

### Development of the FFQ

The FFQ was developed using food items recorded in a single 24-hour diet recall of adults from the Malaysian Adult Nutrition Survey (MANS) 2014^(14)^ and adolescents from the Adolescent Nutrition Survey (ANS) 2017.^(15)^ Both were cross-sectional, nationwide surveys utilizing cluster sampling and proportionate allocation to ensure population representativeness. These dietary recalls provided granular data on brand names, meal occasions, and preparation methods. Respondents estimated portion sizes using a food album and standardized household measures, including cups, spoons, and bowls. Energy and nutrient intakes were calculated primarily using the Malaysian Food Composition Database. We extracted a total of 55,000 food items and categorized them based on their primary ingredients, nutrient profiles, and conceptual similarities. To ensure the inclusion of major nutrient contributors across Malaysia’s diverse ethnicity, we ranked food items separately for Malay, Chinese, Indian, and the indigenous groups of Sabah and Sarawak. We calculated the percentage of micronutrient consumption for each food group using Block equations. Ultimately, food items contributing at least 90% to the total intake of energy and selected nutrients were selected for the final FFQ food list.^(17–19)^

Content validity was established through an expert panel’s evaluation of the FFQ layout and food list. The expert panel included researchers with extensive experience in FFQ development, portion-size estimation, and dietary assessment within national surveys and cohort studies. To account for Malaysia’s diverse culinary landscape, we included sub-type items to capture variations in cooking methods, gravies, and sweeteners. Furthermore, we explicitly listed items of public health significance, such as whole grains, legumes, and sugar-sweetened beverages (including packaged, premixed, and artisanal versions) as separate entries. At the request of stakeholders, common fruits and vegetables were also itemized.

**Figure 1.**
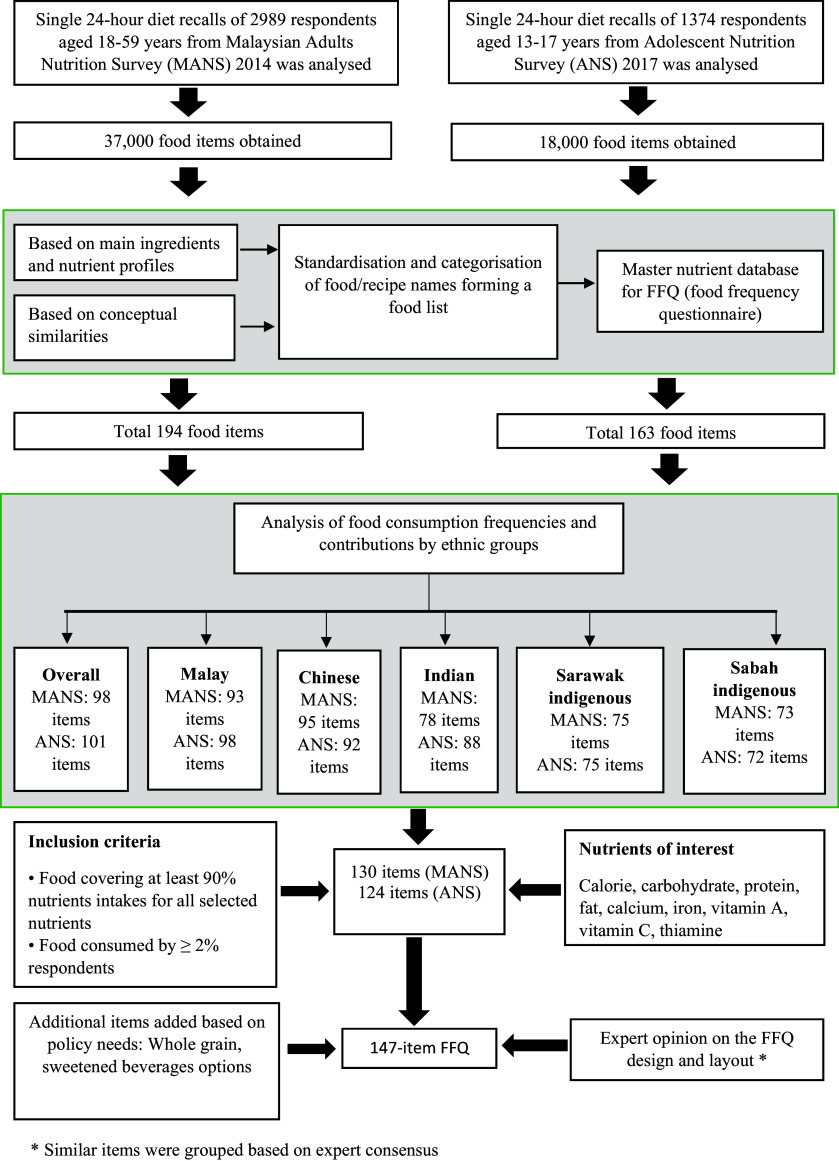
Development of the semi-quantitative food frequency questionnaire.

The draft instrument underwent pretesting to identify usability issues and assess respondent burden. The finalized FFQ comprised of 147 items categorized into 13 groups: (a) cereals and products, (b) meat and products, (c) fish and seafood, (d) eggs, (e) legumes and products, (f) milk and dairy, (g) vegetables, (h) fruits, (i) confectionery, (j) beverages, (k) soup or gravies, (l) spreads, and (m) seasonings and flavourings (Appendix). To facilitate visual portion-size estimation, we developed a corresponding food photo album. Furthermore, a comprehensive nutrient database was compiled using data from the Malaysian and Singaporean Food Composition Databases, supplemented by the Atlas of Food Exchanges and Portion Sizes. The integrated workflow for the FFQ development is illustrated in Figure 1.

### Data collection for validation study

This FFQ validation study employed a cross-sectional design. We recruited approximately 160 government community nutritionists from 13 states and three federal territories to serve as interviewers. To ensure high data quality and inter-rater reliability, we conducted decentralized training sessions by geographical zone. All interviewers were provided with a comprehensive standardized manual containing the study protocol, structured questionnaires, food coding systems, and detailed food descriptions. Training sessions included standardized briefings, mock interview exercises, and competency assessments to ensure uniform administration of the FFQ and 3DRR. We enrolled a total of 400 participants, comprising 200 adolescents and 200 adults. Respondents were selected using a purposive sampling strategy with proportionate allocation based on age and ethnicity to ensure a representative cross-section of Malaysia’s major ethnic groups (Malay, Chinese, Indian, and the indigenous populations of Sabah and Sarawak). Purposive sampling was employed over random sampling due to its feasibility in reaching specific ethnic and age group quotas. Interviews were conducted in-person at the respondents’ homes or workplaces to ensure a comfortable and private environment. Eligible participants included healthy Malaysian citizens aged 10–59 years. We excluded individuals who were pregnant, illiterate, or currently following a medically prescribed diet. Furthermore, those with serious chronic illnesses or physical conditions that could significantly alter habitual dietary patterns were also excluded from the study.

Consistent with established dietary assessment protocols for paediatric populations, interviews for children aged 10–14 years were parent-assisted. Research suggests that while children begin to self-report reliably around age 10, parental input remains essential for quantifying portion sizes and identifying ingredients in complex mixed dishes.^(20)^ Furthermore, the interviewer-administered format allowed trained nutritionists to guide the younger respondents through the survey, providing clarifications and using visual aids to reduce respondent burden and maintain engagement.^(21)^ Although the FFQ food list were collected from school-based surveys of adolescents aged 13–17 years, the age range was expanded to 10–17 years to align with the World Health Organization’s (WHO) official classification of adolescence. This ensures the tool’s utility across the full developmental spectrum of the adolescent population in Malaysia.

**Figure 2.**
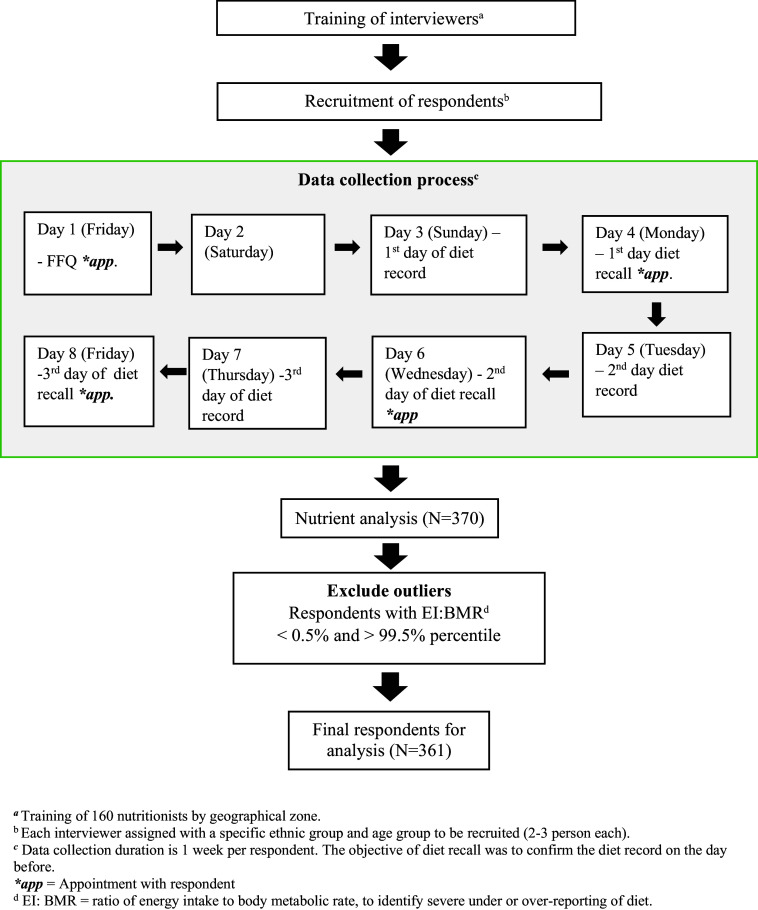
Food frequency questionnaire (FFQ) validation process flowchart.

Data collection proceeded in two distinct phases between September 1 and October 1, 2021. First, nutritionists administered the 147-item FFQ to capture habitual intake frequency, portion sizes, and cooking methods over the past month. To facilitate accurate portion-size estimation, interviewers utilized standardized household measures (cups, bowls, and spoons) alongside a dedicated food photo album. Two days after the FFQ administration, the second phase began with the initiation of the three-day dietary record and recall (3DRR), which served as the reference method. Respondents recorded their dietary intake over three non-consecutive days (two weekdays and one weekend day), providing detailed descriptions of food items, brands, and preparation methods. These records were subsequently reviewed by nutritionists during a follow-up recall session to ensure accuracy and resolve any ambiguities on portion sizes and food items. Additionally, anthropometric measurements were performed by nutritionists at the clinics. All completed records were audited by regional supervisors for completeness and coding accuracy before being centralized for nutrient analysis (Figure 2).

### Analysis of nutrients

Nutrient intake for each participant from the 3DRR was analysed using Nutritionist Pro™. For the FFQ, daily intake for each food item was derived by converting the reported consumption frequency into a daily equivalent using standardized conversion factors. We then calculated the nutrient content for each item by multiplying its daily intake (in grams) by the nutrient values obtained from the Malaysian Food Composition Database and the Singapore Food Composition Database. For food items absent from these databases, nutritional labels and standardized recipes served as supplementary references. We excluded nutrients from dietary supplements to ensure the analysis reflected only habitual food intake. Finally, total daily energy and nutrient intakes were determined by summing the values across all 147 food items. This process ensured a comprehensive and consistent comparison between the FFQ and the 3DRR reference method. The formulas used were:

Daily intake (grams) = Frequency of intake (conversion factor) × Serving size × Total number of servings × Weight of one serving

*Frequency of intake refers to how often the respondent consumes the food (e.g. daily, weekly, or monthly). The conversion factor is used to standardize the frequency into daily values (Daily intake = ×1; Weekly intake = ×1/7; Monthly intake: ×1/30)*

*Serving size: The amount of food typically consumed in one sitting (based on reference portion sizes).*

*Total number of servings: the number of servings consumed at each eating occasion*

*Weight of one serving: The standard weight (in grams) of one serving of the specific food item, based on food composition tables or dietary guidelines.*

For each nutrient of each respondent,

Nutrient value (per food item) = Daily intake (grams) × Nutrient content per gram

Total daily nutrient intake = Sum of nutrient values from all food items

Data preprocessing began with a rigorous cleaning phase to ensure the integrity of the dietary records. To minimize the underreporting and data missingness inherent in self-recorded diaries of the preceding day, each dietary record was reviewed via a face-to-face 24-hour recall interview to clarify portion sizes and identify omitted food items. As such, incomplete information on socio-demography and dietary intake were kept to minimal. Records with missing sociodemographic information were excluded from the final analysis to ensure a high-quality dataset. Maintaining complete profiles for all respondents was essential to establish a rigorous standard for comparison and to ensure the accuracy of the validation results. Any non-recorded intake was assumed as ‘zero-intake’. To ensure the physiological plausibility of the reported intakes, we identified outliers using the Epic-Norfolk method.^(22)^ The ratio of energy intake to basal metabolic rate (EI:BMR) below the 0.5th percentile or above the 99.5th percentile were classified as implausible and subsequently excluded. The BMR for each respondent was calculated using validated, gender-specific formulas for the Malaysian population.^(23)^ The comprehensive workflow of the validation study, from interviewer training to the final sample size (*n* = 361) available for analysis, is depicted in Figure 2.

### Statistical analysis

Sociodemographic characteristics and body mass index (BMI) were described using frequencies and percentages for categorical variables and means with standard deviations (SDs) for continuous variables. Due to skewed distributions, energy and nutrient intakes from the FFQ and 3DRR were presented as medians and interquartile ranges (IQR; Q1, Q3). We utilized the Wilcoxon signed-rank test to assess differences in nutrient distribution between the two methods. Spearman’s correlation coefficients were calculated to estimate the relative validity of the FFQ against the 3DRR, with thresholds defined as poor (<0.2), acceptable (0.2–0.5), and good (≥0.5).^(24)^ To account for variations in total caloric intake, nutrient intakes were energy-adjusted using the residual method. In this approach, nutrient intake was regressed on total energy intake; the resulting residuals represent energy-adjusted values independent of total caloric consumption. An increase in the correlation coefficient after energy adjustment suggested the nutrient was significantly linked to total energy intake, whereas a decrease indicated that the initial correlation was likely inflated by energy consumption.

To evaluate ranking agreement, we performed a cross-classification of quartiles for energy and all nutrient intakes. Participants were categorized into quartiles to determine the proportion of individuals classified into the same, adjacent, or opposite (extreme) quartiles by both methods. In accordance with established criteria, a proportion of ≤10% in the opposite quartile was considered to represent good agreement.^(24)^ Bland–Altman plots were utilized to assess the level of agreement and the presence of systematic bias for energy and macronutrients. These plots displayed the difference between the two methods (FFQ - 3DRR) on the y-axis against their mean ((FFQ + 3DRR)/2) on the x-axis. The mean difference indicated whether the FFQ tended to over-or underestimate intake, while the limits of agreement (LOA) (mean ± 1.96 SD) reflected the degree of precision. A random distribution of data points around the zero-mean difference line indicated an absence of proportional bias, whereas consistent clustering above or below the line suggested that the methods were not interchangeable. All statistical analyses were performed using IBM SPSS Statistics version 23, and all statistical significance was set at *p* < 0.05.

## Results

A total of 361 participants were included in the final analysis, comprising equal proportions of males and females, and of adolescents and adults. The ethnic distribution (Malay, Chinese, Indian, and the indigenous populations of Sabah and Sarawak) was well balanced, with each group representing between 17.6% and 25.6% of the total sample. Among the adult cohort, 64.5% attained tertiary education, and 76.0% were currently employed. Geographically, 61.0% of respondents resided in Peninsular Malaysia, while 39.0% were from East Malaysia. When stratified by age, the mean BMI was 20.86 ± 5.26 kg/m^2^ for adolescents (10–17 years) and 25.83 ± 4.93 kg/m^2^ for adults (18–59 years). These values were statistically comparable within their respective age-standardized growth and health categories (Table 1).

**Table 1.** Characteristics of respondents (*N* = 361)

Characteristics	All (N = 361)	Adolescent (*N* = 177)	Adult (*N* = 184)
	*n* (%)*		
Gender			
Male	179 (49.6)	91 (51.1)	88 (48.1)
Female	182 (50.4)	87 (48.9)	95 (51.9)
Age (Mean ± SD), years	25 ± 14	13 ± 2	36 ±12
Ethnicity			
Malay	91 (25.2)	48 (27.0)	43 (23.5)
Chinese	81 (22.4)	42 (23.6)	39 (21.3)
Indian	57 (15.8)	26 (14.6)	31 (16.9)
Sabah Indigenous	69 (19.1)	33 (18.5)	36 (19.7)
Sarawak Indigenous	62 (17.2)	29 (16.3)	33 (18.0)
States			
Johor	17 (4.7)	9 (5.1)	8 (4.4)
Kedah	15 (4.2)	7 (3.9)	8 (4.4)
Kelantan	20 (5.5)	10 (5.6)	10 (5.5)
Melaka	14 (3.9)	7 (3.9)	7 (3.8)
Negeri Sembilan	18 (5.0)	9 (5.1)	9 (4.9)
Pahang	19 (5.3)	10 (5.6)	9 (4.9)
Pulau Pinang	20 (5.5)	10 (5.6)	10 (5.5)
Perak	18 (5.0)	8 (4.5)	10 (5.5)
Perlis	10 (2.8)	5 (2.8)	5 (2.7)
Selangor	27 (7.5)	13 (7.3)	14 (7.7)
Terengganu	20 (5.5)	11 (6.2)	9 (4.9)
Sabah and Labuan	70 (19.4)	34 (19.1)	36 (19.7)
Sarawak	73 (20.2)	35 (19.7)	38 (20.8)
KL/Putrajaya	20 (5.5)	10 (5.6)	10 (5.5)
Education level			
Primary/unknown	201 (55.8)	177 (100)	24 (13.1)
Secondary	41 (11.4)	0	41 (22.4)
Tertiary	118 (32.8)	0	118 (64.5)
Employment status			
Yes	139 (38.5)	0	139 (76.0)
No	222 (61.5)	177 (100)	44 (24.0)
Body mass index, Mean ± SD; kg/m^2^	23.5 ± 5.8	20.8 ± 5.4	26.1 ± 5.0

* Figures are *n* (%), unless indicated otherwise.

Median energy intake reported from the 3DRR was 1,768 kcal (48.4% carbohydrate, 15.6% protein, 34.6% fat), whereas the FFQ yielded a significantly higher median of 2,285 kcal (66.2% carbohydrate, 17.0% protein, 25.6% fat). Apart from fats, the FFQ significantly overestimated the intake of all nutrients compared to the 3DRR (*p* < 0.05). The Spearman correlation coefficient for energy was 0.31, with a mean of 0.27 for macronutrients. Most nutrient correlations exceeded the acceptable threshold of 0.20, except for protein. Following energy adjustment via the residual method, correlations for fats, Vitamin C, and thiamine increased, while other nutrients were attenuated. Cross-classification analysis revealed that over 70% of respondents were correctly classified into the same or adjacent quartiles for most nutrients. Extreme misclassification (>10%) occurred only for protein intake, whilst the highest agreement was observed for carbohydrate at 38.0% (Table 2).

**Table 2.** Nutrient content and differences between 3DRR and FFQ of all respondents (*N* = 361)

Nutrient	3DRR	FFQ	Spearman’s correlation, *r*		Cross-classification analysis, (%)		
	Median (Q1, Q3)	Median (Q1, Q3)	Crude	Energy adjusted^a^	Same quartile	Same and adjacent quartile	Extreme quartile^b^
Energy (kcal)	1768 (1385, 2292)	2285 (1678, 3225)***	0.31	–	35.8	72.8	8.6
CHO (g)	214 (159, 285)	378 (273, 509)***	0.35	0.20	38.0	75.1	6.4
Protein (g)	69 (54, 91)	97 (67, 143)***	0.18	0.14	30.2	71.2	11.1
Fat (g)	68 (52, 92)	65 (45, 102)	0.28	0.23	33.2	73.1	7.8
Vit C (mg)	46 (26, 75)	147.0 (84, 228)***	0.33	0.38	34.9	76.2	7.8
Thiamine (ug)	0.69 (0.50, 0.89)	1.60 (1.24, 2.14)***	0.24	0.34	30.5	68.7	6.9
Calcium (mg)	466 (330, 631)	1288 (658, 2097)***	0.25	0.22	32.7	70.9	6.4
Iron (mg)	14.2 (10.2, 18.9)	21.3 (15.6, 29.7)***	0.22	0.19	28.8	70.4	7.8

*** *p* < 0.001 of Wilcoxon signed-rank test.

a Energy-adjusted correlation coefficients of nutrients are independent of energy in 3DRR and FFQ.

b Being classified from one extreme category to another extreme category.

**Figure 3.**
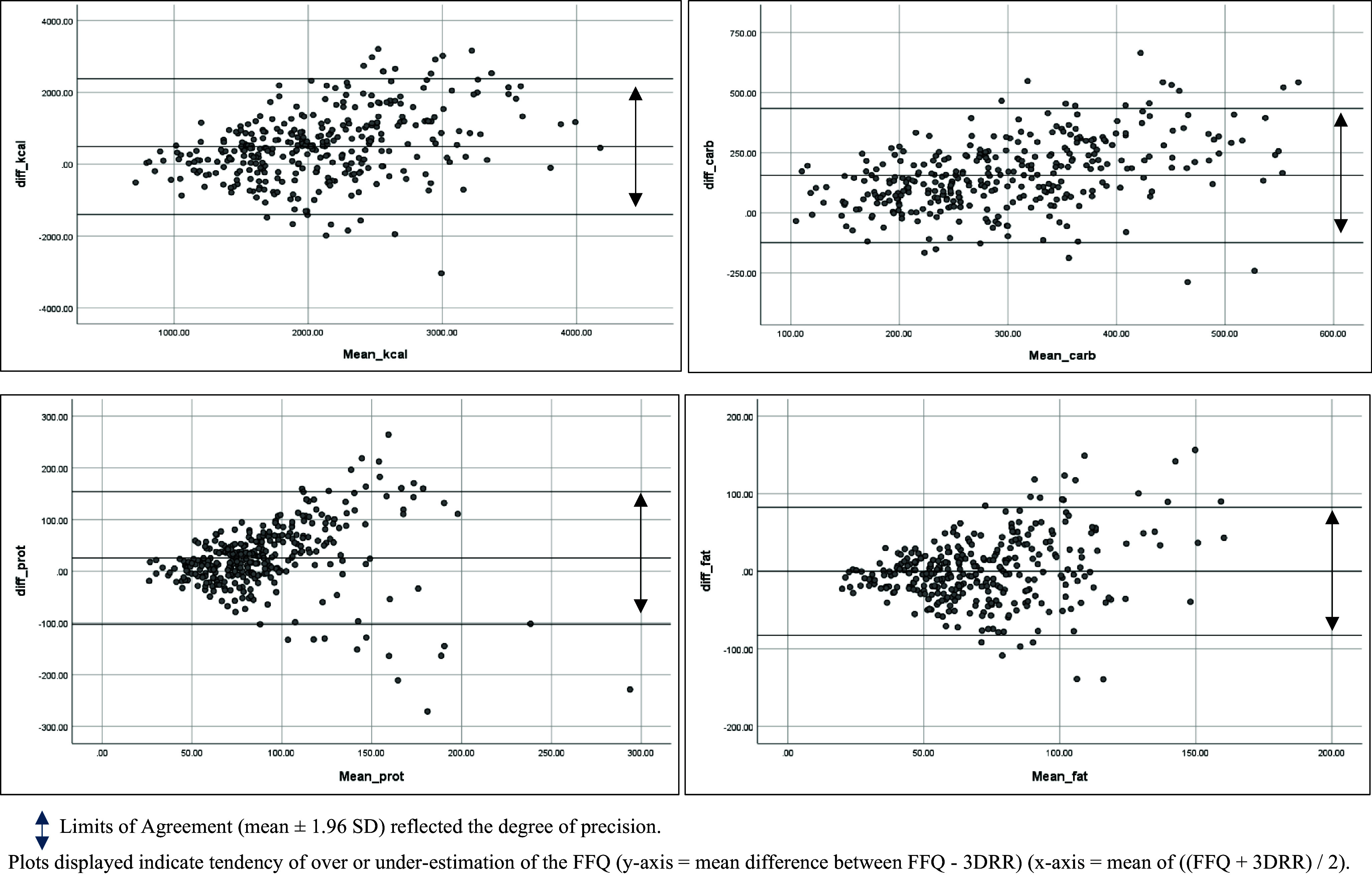
Bland–Altman plots of energy and macronutrients.

Bland–Altman plots for energy and macronutrients demonstrated that majority of data points fell within the 95% Limits of Agreement (LOA), suggesting acceptable individual consistency between the two methods. For all macronutrients, the mean difference line either clustered closely around zero, or the data points were symmetrically distributed within the 95% LOA. This suggests that the FFQ did not consistently over-or underestimate intakes relative to the 3DRR, confirming that the two methods provide comparable estimates for the population (Figure 3).

The Spearman correlation coefficients between the 3DRR and the FFQ were lower among adolescents compared to adults, particularly protein and iron intake. Across both groups, vitamin C and thiamine consistently demonstrated the most robust correlations after energy adjustment (Table 3).

**Table 3.** Correlation coefficients between 3DRR and FFQ of adults and adolescents

	Adults (*n* = 184)		Adolescents (*n* = 177)	
Nutrient	Spearman’s correlation coefficients, *r*			
	Crude	Energy adjusted*	Crude	Energy adjusted*
Energy (kcal)	0.38	–	0.23	–
CHO (g)	0.40	0.18	0.30	0.26
Protein (g)	0.31	0.18	0.04	0.12
Fat (g)	0.37	0.27	0.22	0.16
Vit C (mg)	0.35	0.38	0.31	0.38
Thiamine (ug)	0.32	0.34	0.17	0.34
Calcium (mg)	0.30	0.24	0.20	0.19
Iron (mg)	0.28	0.32	0.17	0.09

* Energy-adjusted correlation coefficients of nutrients are independent of energy values in both 3DRR and FFQ.

## Discussion

The primary objective of this study was to develop and validate a 147-item FFQ, capable of assessing habitual dietary intake among Malaysian adolescents and adults to inform national nutrition policy. Our findings demonstrated that the FFQ possessed a low to average relative validity against the 3DRR reference method for energy and macronutrients, with minimal extreme quartile misclassification. The relative validity for energy aligns with coefficient ranges reported in similar validation studies across Southeast Asia, which have typically yielded correlations between 0.20 and 0.50.^(12, 25–30)^ Similarly, in a study among Ukrainian adult population, the correlation for most nutrients ranged from 0.21–0.40.^(31)^ Although previously validated FFQs in Malaysia had reported higher correlation coefficients, those instruments were restricted to specific population subgroups, limiting their national-level applicability.^(11, 32–34)^ Validated FFQ from other countries with higher correlation coefficients were also those with non-diverse culture food intake such as in China, Italy, Japan and Brunei.^(33–37)^ This reflects the inherent challenges of dietary recall and the complexity of multi-ingredient Asian dishes. Crucially, the low rate of extreme quartile misclassification and the absence of proportional bias support the suitability of this FFQ for large-scale epidemiological surveillance across urban and rural locations.

Lower correlation coefficients were reported for protein and iron, suggesting greater challenges in capturing the intake of animal-based food sources. This could reflect a substantial variability in protein consumption and the complexities of estimating portion sizes in mixed dishes. In the FFQ, portions were estimated using serving sizes such as ‘pieces’ for poultry, ‘spoons’ for other meats, and ‘medium size’ for seafood. Such estimations are inherently subjective despite guided pictorials provided. Moreover, cooking methods and individual perceptions further alter portion sizes. Although previous literature had suggested that portion-size estimation does not significantly impact FFQ results, those studies were conducted in a more homogeneous population with less dietary variation.^(38–40)^ In the Malaysian context, the diversity of mixed dishes makes portion estimation particularly challenging. In addition, potential underreporting may be attributed to social desirability bias, where participants under-report meat intake due to its perceived health implications.^(41)^ Interestingly, vitamin C and thiamine showed the most stable and highest correlations. This suggests that fruits and cereals of which these nutrients are likely derived were easily identifiable, recalled and quantified more reliably than items found in a variety of mixed dishes. Future research should, therefore, focus on refining portion-size estimation for protein-rich items within multi-cultural dietary assessments.

The correlation coefficients observed in this study were generally weaker among adolescents compared to adults. As the FFQ requires respondents to recall and estimate their average food intake over a one-month period, adolescents with sporadic eating habits may have greater difficulty with retrospective assessments.^(30)^ In contrast, adults are more likely to possess a more consistent understanding of portion sizes and food ingredients, which may contribute to better reporting accuracy. Nevertheless, utilizing a standardized FFQ that caters to both adults and adolescents remains essential to national nutrition surveillance. This is to ensure that findings are comparable and could provide a unified evidence base to inform the development of national dietary guidelines. Future research should consider transitioning to digital FFQs that utilize automated skip-logic and provide instant definitions or examples for complex mixed dishes. Furthermore, incorporating graduated portion-size imagery can facilitate a more precise self-reporting by helping participants better visualize and match their actual consumption.

## Strengths and limitations

This study possesses several strengths, particularly the multisectoral collaboration between policymakers, academic experts, and community nutritionists involved in the development of the FFQ. This partnership ensured that the FFQ was not only scientifically rigorous but also practically aligned with public health needs. Although the food list was primarily derived from previous survey data, expert consensus allowed for the rational inclusion of current trends, alongside items of public health significance. Furthermore, while the 147-item list exceeded the common 130-item threshold for respondent burden,^(30,42)^ the instrument remained efficient, with an average administration time of 30 minutes. Interestingly, compared to the 3DRR, the carbohydrate intake per total energy reported in the FFQ aligned more closely with current recommended intake of 60% (FFQ: 66% vs 3DRR: 48.4%), suggesting that the 3DRR may have under-reported intakes. Despite the higher median nutrient reported in the FFQ, the Bland–Altman analyses showed no consistent over-reporting. This indicated that the extensive food list aided recall rather than artificially inflating the data. Moreover, the inclusion of respondents from both urban and rural areas across the entire country ensured extensive geographical and demographic diversity. Consequently, this study represents one of the most comprehensive dietary validation efforts conducted within the region.

However, several limitations must be acknowledged. The study relied exclusively on the 3DRR as the reference standard without the inclusion of objective biomarkers. Although objective biomarkers may provide a more precise assessment of certain nutrient intake and reduce reliance on self-reported data, their inclusion was logistically and financially prohibitive for this study. Furthermore, biomarkers generally reflect short-term intake or physiological status for selected nutrients only, whereas dietary assessment methods such as the FFQ and 3DRR could capture broader habitual dietary patterns and food consumption behaviours. While the 3DRR is widely recognized as a reference method for dietary validation studies, it remains susceptible to recall bias, underreporting, and day-to-day dietary variation, which may affect the accuracy of intake estimation and attenuate correlations with the FFQ. Therefore, the findings of this study should be interpreted within the context of self-reported dietary assessment methods, acknowledging that some degree of measurement error is unavoidable in nutritional epidemiology.

Furthermore, inter-interviewer variability may have contributed to measurement bias. The involvement of 160 interviewers was logistically necessary to ensure adequate coverage of a geographically diverse population. However, differences in interviewing techniques and probing methods may have influenced participants’ responses. Nevertheless, we have not found any systematic measurement bias between interviewers in the analysis. Additionally, the nutrient databases used lacked complete information for certain food items, particularly complex mixed dishes and cooked meals commonly consumed in Malaysia. As a result, some nutrient estimates were robust approximations rather than precise absolute measurements. Despite these limitations, the validated FFQ remains a practical and low-burden tool for nationwide dietary surveillance, with strong potential to generate valuable data for monitoring population dietary patterns and informing diet-related national policies and interventions.

## Conclusion

In conclusion, the semi-quantitative FFQ developed in this study demonstrated acceptable relative validity and robust ranking agreement for assessing habitual dietary intake across Malaysia’s diverse, multi-ethnic population. The data derived from this tool will be instrumental in monitoring dietary trends and evaluating population-level adherence to the Malaysian Dietary Guidelines. Moving forward, integrating this FFQ into digital platforms and refining portion-size estimation for complex mixed dishes will further enhance its precision. Ultimately, this validated FFQ provides the evidence-based foundation necessary to design targeted public health interventions and policies aimed at mitigating the rising burden of diet-related chronic diseases in Malaysia.

## Supporting information

10.1017/jns.2026.10119.sm001 Wong et al. supplementary material Wong et al. supplementary material

## Supplementary material

The supplementary material for this article can be found at https://doi.org/10.1017/jns.2026.10119.
