# Supplementary material for: Development and validation of a food frequency questionnaire for national nutrition surveillance among Malaysian adolescents and adults
Source: J Nutr Sci. 2026 Jul 1;15:e48. doi: 10.1017/jns.2026.10119 (PMC13323619; doi:10.1017/jns.2026.10119)
Supplement: Wong et al. supplementary material [file S2048679026101190sup001.docx]

**Appendix:** List of food items in the FFQ

| 1 | White rice |
| --- | --- |
| 2 | Glutinous rice |
| 3 | Wholegrain rice (eg. brown/ red/ black) |
| 4 | Fried rice |
| 5 | Nasi lemak |
| 6 | Chicken rice |
| 7 | Flavoured rice |
| 8 | Rice porridge |
| 9 | Sushi |
| 10 | Wheat-based noodles |
| 11 | Rice-based noodles |
| 12 | Instant noodles |
| 13 | Pasta |
| 14 | Roti canai/ paratha |
| 15 | Flavoured roti canai |
| 16 | Tosai |
| 17 | Capati |
| 18 | Bread /bun, Plain white |
| 19 | Bread / Bun, Wholegrain |
| 20 | Sweet bread/bun (filled, cream, flavoured) |
| 21 | Savoury bread/bun (filled, flavoured) |
| 22 | Burger |
| 23 | Sandwich |
| 24 | Pizza |
| 25 | Breakfast cereals |
| 26 | Cereals grain prepared with water |
| 27 | Corn |
| 28 | Butter/ margarine |
| 29 | Chocolate spread |
| 30 | Peanut butter |
| 31 | Coconut jam |
| 32 | Jam |
| 33 | Chicken |
|  | A Fried |
|  | B Roasted/ grill |
|  | C Cooked with coconut milk |
|  | D Cooked without coconut milk |
|  | E Soup/ simmered/ steamed |
| 34 | Beef |
|  | A Fried |
|  | B Roasted/ grill |
|  | C Cooked with coconut milk |
|  | D Cooked without coconut milk |
|  | E Soup/ simmered/ steamed |
| 35 | Pork |
|  | A Fried |
|  | B Roasted/ grill |
|  | C Cooked with coconut milk |
|  | D Cooked without coconut milk |
|  | E Soup/ simmered/ steamed |
| 36 | Mutton |
| 37 | Duck |
| 38 | Internal organs |
| 39 | Processed meat (Sausage/Nugget/Burger meats/Luncheon meat) |
| 40 | Marine fish |
|  | A Fried |
|  | B Roasted/grill |
|  | C Cooked with coconut milk |
|  | D Cooked without coconut milk |
|  | E Soup/ simmered/ steamed |
| 41 | Freshwater fish |
|  | A Fried |
|  | B Roasted/ grill |
|  | C Cooked with coconut milk |
|  | D Cooked without coconut milk |
|  | E Soup/ simmered/ steamed |
| 42 | Prawn |
|  | A Fried |
|  | B Roasted/ grill |
|  | C Cooked with coconut milk |
|  | D Cooked without coconut milk |
|  | E Soup/ simmered/ steamed |
| 43 | Squid |
|  | A Fried |
|  | B Roasted/ grill |
|  | C Cooked with coconut milk |
|  | D Cooked without coconut milk |
|  | E Soup/ simmered/ steamed |
| 44 | Crab |
| 45 | Shellfish |
| 46 | Anchovy |
|  | A Fried |
|  | B Cooked with coconut milk |
|  | C Cooked without coconut milk |
|  | D Soup/ simmered/ steamed |
| 47 | Canned fish |
| 48 | Dried/ Salted fish |
| 49 | Dried cuttlefish |
| 50 | Seafood products/ surimi |
| 51 | Keropok lekor |
| 52 | Sata ikan |
| 53 | Hen/quail/duck eggs |
|  | A Fried |
|  | B Boiled |
|  | C Cooked with coconut milk |
|  | D Cooked without coconut milk |
|  | E Soup/ simmered/ steamed |
| 54 | Salted eggs |
| 55 | Green leafy vegetables |
| 56 | Ladies finger |
| 57 | Lettuce |
| 58 | Tomato |
| 59 | Local fresh salads |
| 60 | Legumes vegetables |
| 61 | Bean sprout |
| 62 | Tubers |
| 63 | Cruciferous vegetables |
| 64 | Fruit vegetables |
| 65 | Root vegetables |
| 66 | Brinjal/ eggplant |
| 67 | Mushrooms (moist & dried) |
| 68 | Salted/dried vegetables |
| 69 | Lotus root |
| 70 | Fries |
| 71 | Coleslaw |
| 72 | Groundnuts |
| 73 | Other Groundnuts |
| 74 | Nuts/ Legumes |
| 75 | Tofu |
| 76 | Tempe / Fermented soybeans |
| 77 | Dhal gravy |
| 78 | Sardine gravy |
| 79 | Chutney |
| 80 | Gravy with coconut milk |
| 81 | Gravy without coconut milk |
| 82 | Soup |
| 83 | Apple |
| 84 | Orange |
| 85 | Pear |
| 86 | Papaya |
| 87 | Watermelon |
| 88 | Honeydew |
| 89 | Guava |
| 90 | Mango |
| 91 | Rambutan |
| 92 | Banana |
| 93 | Jackfruit |
| 94 | Pineapple |
| 95 | Longan |
| 96 | Durian |
| 97 | Water apple |
| 98 | Canned fruits |
| 99 | Dried fruits |
| 100 | Non-flavoured milk |
| 101 | Flavoured milk |
| 102 | Powdered milk |
| 103 | Evaporated milk |
| 104 | Skimmed milk |
| 105 | Yogurt |
| 106 | Cheese |
| 107 | Plain water |
| 108 | Tea |
|  | A. Self-prepared |
|  | Ai. Sugar |
|  | Aii. Sweetened condensed milk |
|  | B. Store-prepared |
|  | Bi. Sweetened |
|  | Bii. Unsweetened |
|  | C. Pre-mixed powders |
|  | D. Ready-to-drink |
| 109 | Coffee |
|  | A. Self-prepared |
|  | Ai. Sugar |
|  | Aii. Sweetened condensed milk |
|  | B. Store-prepared |
|  | Bi. Sweetened |
|  | Bii. Unsweetened |
|  | C. Pre-mixed powders |
|  | D. Ready-to-drink |
| 110 | Malted / Chocolate drinks |
|  | A. Self-prepared |
|  | Ai. Sugar |
|  | Aii. Sweetened condensed milk |
|  | B. Store-prepared |
|  | C. Pre-mixed powders |
|  | D. Ready-to-drink |
| 111 | Cordial drinks |
|  | A. Self-prepared |
|  | Ai. Sugar |
|  | Aii. Sweetened condensed milk |
|  | B. Store-prepared |
| 112 | Other pre-mixed drinks (2in1, 3in1) |
|  | A. Sugar |
|  | B. Sweetened condensed milk |
| 113 | Fresh fruit juices |
|  | A. Self-prepared |
|  | Ai. Sugar |
|  | Aii. Sweetened condensed milk |
|  | B. Store-prepared |
|  | C. Ready to drink fruit-flavoured juice |
|  | D. Ready to drink 100% fruit Juice |
|  | E. Ready to drink vegetable juice |
| 114 | A. Carbonated drink |
|  | B. Isotonic or sport drinks |
| 115 | Energy drinks |
| 116 | Soymilk (or other Plant-based milk) |
| 117 | Other Ready-to-drink beverages |
| 118 | Yoghurt drinks |
| 119 | Cultured milk |
| 120 | Drinks with BOBA |
| 121 | Other non-listed sweetened-beverages |
| 122 | Alcoholic beverages |
| 123 | Sweet fried kuih |
| 124 | Sweet non-fried kuih including sweetened porridge |
| 125 | Savoury fried kuih |
| 126 | Savoury non-fried kuih |
| 127 | Cream crackers |
| 128 | Flavoured/cream/filled biscuits |
| 129 | Cake |
|  | A. Buttercake/muffin/ cupcake |
|  | B. Sponge cake |
|  | C. Cheesecake |
| 130 | Fish/prawn/squid/crab crackers |
| 131 | Snacks/crackers |
| 132 | Candy/chewing gum |
| 133 | Chocolate |
| 134 | Ice-cream |
| 135 | Jelly/ pudding/ agar-agar/ custard |
| 136 | Ice-blended dessert |
| 137 | Table salt |
| 138 | Chilli/ tomato sauce |
| 139 | Mayonnaise |
| 140 | Tartar/thousand island/ cheese sauce |
| 141 | Fish/squid/oyster sauce |
| 142 | Soy sauce |
| 143 | Shrimp paste |
| 144 | Cencaluk |
| 145 | Budu |
| 146 | Asam boi powder |
| 147 | Sugar/ Honey / Other sweeteners |
